# MRI-based radiomics value for predicting the survival of patients with locally advanced cervical squamous cell cancer treated with concurrent chemoradiotherapy

**DOI:** 10.1186/s40644-022-00474-2

**Published:** 2022-07-16

**Authors:** Xiaomiao Zhang, Jingwei Zhao, Qi Zhang, Sicong Wang, Jieying Zhang, Jusheng An, Lizhi Xie, Xiaoduo Yu, Xinming Zhao

**Affiliations:** 1 Department of Radiology, National Cancer Center/National Clinical Research Center for Cancer/Cancer Hospital, Chinese Academy of Medical Sciences and Peking Union Medical College, Beijing, 100021 China; 2 GE Healthcare, MR Research, Beijing, China; 3 Department of Gynecologic Oncology, National Cancer Center/National Clinical Research Center for Cancer/Cancer Hospital, Chinese Academy of Medical Sciences and Peking Union Medical College, Beijing, 100021 China

**Keywords:** Cervical squamous cell cancer, FIGO stage, Radiomics, Progression-free survival, Overall survival

## Abstract

**Background:**

To investigate the magnetic resonance imaging (MRI)-based radiomics value in predicting the survival of patients with locally advanced cervical squamous cell cancer (LACSC) treated with concurrent chemoradiotherapy (CCRT).

**Methods:**

A total of 185 patients (training group: *n* = 128; testing group: *n* = 57) with LACSC treated with CCRT between January 2014 and December 2018 were retrospectively enrolled in this study. A total of 400 radiomics features were extracted from T2-weighted imaging, apparent diffusion coefficient map, arterial- and delayed-phase contrast-enhanced MRI. Univariate Cox regression and least absolute shrinkage and selection operator Cox regression was applied to select radiomics features and clinical characteristics that could independently predict progression-free survival (PFS) and overall survival (OS). The predictive capability of the prediction model was evaluated using Harrell’s C-index. Nomograms and calibration curves were then generated. Survival curves were generated using the Kaplan-Meier method, and the log-rank test was used for comparison.

**Results:**

The radiomics score achieved significantly better predictive performance for the estimation of PFS (C-index, 0.764 for training and 0.762 for testing) and OS (C-index, 0.793 for training and 0.750 for testing), compared with the 2018 FIGO staging system (C-index for PFS, 0.657 for training and 0.677 for testing; C-index for OS, 0.665 for training and 0.633 for testing) and clinical-predicting model (C-index for PFS, 0.731 for training and 0.725 for testing; C-index for OS, 0.708 for training and 0.693 for testing) (*P* < 0.05). The combined model constructed with T stage, lymph node metastasis position, and radiomics score achieved the best performance for the estimation of PFS (C-index, 0.792 for training and 0.809 for testing) and OS (C-index, 0.822 for training and 0.785 for testing), which were significantly higher than those of the radiomics score (*P* < 0.05).

**Conclusions:**

The MRI-based radiomics score could provide effective information in predicting the PFS and OS in patients with LACSC treated with CCRT. The combined model (including MRI-based radiomics score and clinical characteristics) showed the best prediction performance.

**Supplementary Information:**

The online version contains supplementary material available at 10.1186/s40644-022-00474-2.

## Background

Cervical cancer is the fourth most frequent malignancy in women worldwide, with the fourth highest mortality [1, 2]. Squamous cell carcinoma, which accounts for more than 75% of all cervical cancers, is a major contributor to the overall burden of disease in females [3]. Concurrent chemoradiotherapy (CCRT) remains the standard treatment for patients with locally advanced cervical cancer (LACC) (i.e., those with stage IIB-IVA diagnosed according to the new 2018 International Federation of Gynecology and Obstetrics [FIGO] staging system [4]) according to the latest National Comprehensive Cancer Network Clinical Practice Guidelines in Oncology [5]. Although outcomes in patients with LACC had been improved with multimodality treatment, the overall recurrence rate is 35%, and the median survival after recurrence is still low, only 10–12 months [6, 7]. Moreover, women with recurrent cervical cancer are prone to first-line chemotherapy drug resistance. Thus, predicting cervical cancer outcomes is one of the most challenging tasks. An early and reliable biomarker that can predict patients’ prognosis may help clinicians adjust the treatment plan in time and conduct more intensive follow-up to improve patients’ survival rate and quality of life.

Magnetic resonance imaging (MRI) has excellent multi-planar capability and can produce cross-sectional images of the body with excellent soft-tissue contrast. It is the preferred imaging modality to evaluate locoregional tumor extent. Positron emission tomography-computed tomography (PET/CT) is often used as a supplement for MRI for nodal and distant staging [8]. PET/CT and MRI have been widely used for prognosis evaluation in cervical cancer [9–11]. Compared to conventional PET/CT and MRI parameters, there has been a growing interest in radiomics in recent years. Radiomics is a technology that uses artificial intelligence and machine learning to extract a large set of high-dimensional data from a series of medical images. The extracted features can be used as alternative markers for underlying gene expression patterns and related biological characteristics such as tumor morphology and intratumor heterogeneity [12, 13]. Recent developments of radiomics have shown the potential added value in discriminatory and prognostic evaluation of cervical cancer when using PET/CT and MRI [14–18]. Previous studies found that radiomics features extracted from PET/CT images could more accurately predict survival of cervical cancer patients compared to conventional clinical factors and SUV_max_; yet, these studies had a smaller sample size and PET/CT was less clinically used than MRI [14–16].

MRI plays an essential role in the initial staging of disease, therapeutic strategy, treatment planning, and evaluation of tumor response [19]. Both T2-weighted imaging (T2WI) and diffusion-weighted imaging (DWI) are now recommended for initial staging, assessment of treatment response, and evaluation of recurrence [8]. This study aims to develop radiomics features extracted from pretreatment MRI to predict the progression-free survival (PFS) and overall survival (OS) in patients with locally advanced cervical squamous cell cancer (LACSC) treated with CCRT and then compared their predictive value with clinical characteristics and the new 2018 FIGO staging system.

## Methods

### Research subjects

The institutional review board approved this study and waived the need for informed consent. A retrospective review was performed on patients with cervical cancer treated in our hospital between January 2014 and December 2018. Inclusion criteria were the following: (1) histologically proven cervical squamous cell cancer; (2) FIGO stage IIB to IVA, according to the 2018 FIGO staging system [4]; (3) undergoing pelvic MRI before treatment, including DWI; (4) treated with definitive curative CCRT. Exclusion criteria were: (1) history of other cancers; (2) history of previous chemotherapy or radiotherapy; (3) pretreatment pelvic MRI did not include multi-phase contrast enhanced scannig; (4) images with artifacts; (5) patient that failed to complete the treatment.

**Fig. 1:**
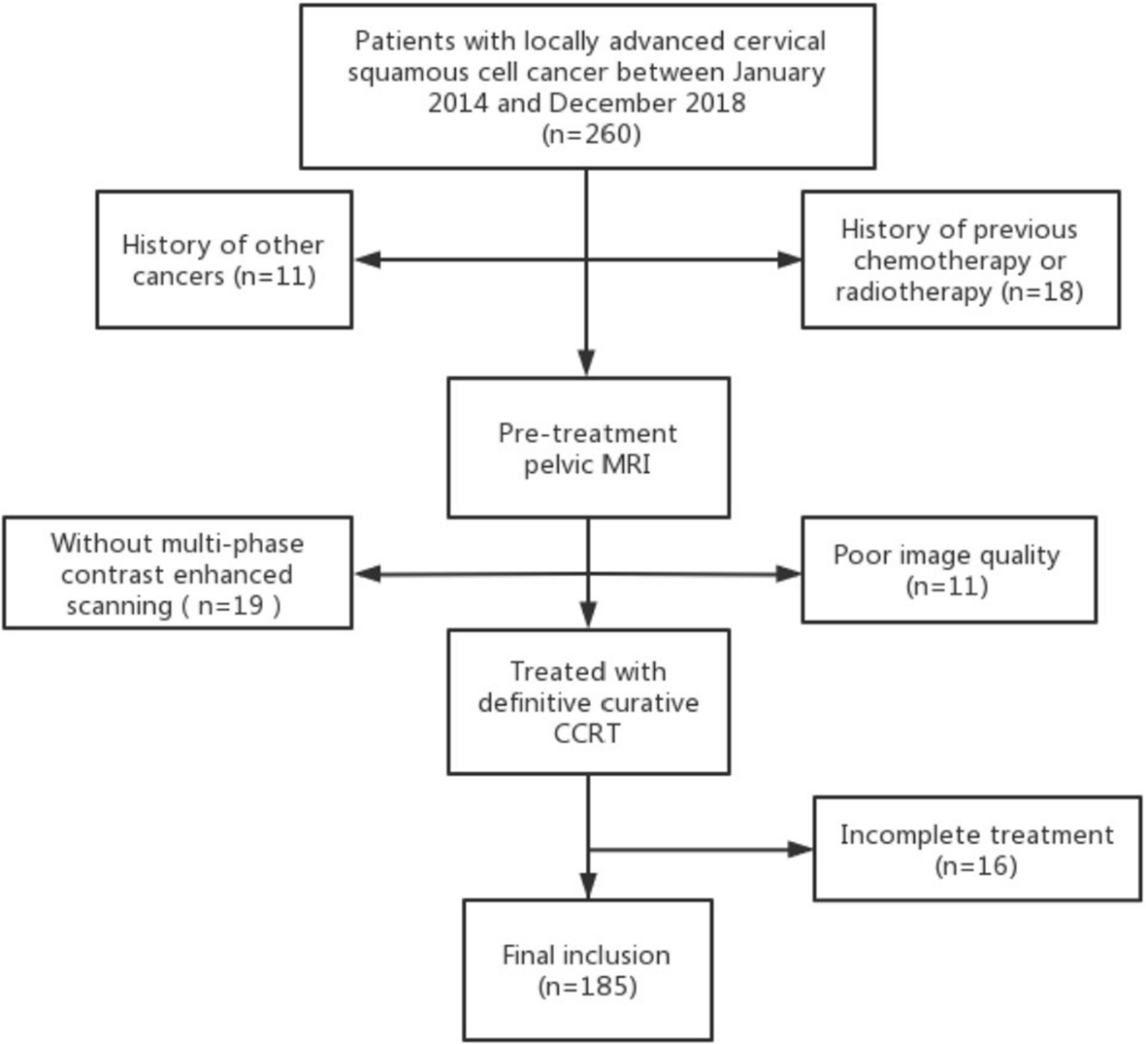
The flowchart of the study cohort

Finally, 185 patients were included in this study (mean age, 52.8 ± 8.8 years; age range, 24–73 years). A flowchart of the study population is shown in Fig. 1. Eligible patients’data were randomly divided into a training group (*n* = 128) and a testing group (*n* = 57) at a ratio of 7:3.

The clinical characteristics included age, body mass index (BMI), squamous cell carcinoma antigen (SCC-Ag), tumor grade (low-grade: well/moderately differentiated; high-grade: poorly differentiated), 2018 FIGO stage, primary tumor invasion (T stage), tumor maximum-diameter, lymph node metastasis (LNM) (number and position [pelvic/para-aortic]). A radiologist with 18 years of experience in gynecological imaging (X. Y.) and a clinician with 20 years of experience in gynecological tumor (J. A.) who were blinded to clinical outcomes of the patients restaged all patients based on clinical records and imaging examination results using the revised 2018 FIGO staging system [4]. The two doctors also classified the T stage in consensus according to TNM 9^th^ edition [20]. Tumor maximum-diameter was determined by the longest diameter measured on T2WI in the sagittal or transverse axial planes. Lymph node with short-axis diameter ≥ 1.0 cm or liquefaction necrosis was assessed as LNM.

### MR techniques

All patients underwent routine contrast-enhanced pelvic MRI before CCRT. MRI acquisitions were performed on two 3.0-T MR imaging units (Discovery MR 750 and Signa Excite HDx, GE Medical System) using an eight-element phased coil with patients in the supine position. Patients with no contraindications received an intramuscular injection of 10 mg raceanisodamine hydrochloride before image acquisition to reduce bowel motion artifacts. DWI was performed using a single-shot spin echo-planar imaging sequence with b values of 0 and 800 s/mm^2^. Sagittal multi-phase contrast enhanced scannig was performed using liver acquisition with volume acceleration-extended volume (LAVA-XV) sequence 15 seconds after an intravenous injection contrast agent (gadodiamide, 0.1 mmol/kg; Omniscan; GE Healthcare, Co. Cork, Ireland) at a rate of 2.0 ml/s, per phase of 15 seconds with a total acquisition time of 105 seconds, followed by 20 mL of normal saline to flush the tubing. Detailed information on the MR sequences is listed in Supplementary Table S1.

### Treatment

All patients received whole pelvic external pelvic beam radiation therapy (EBRT) or extended-field RT to the para-aortic area depending on their work up at 1.8–2.4 Gy daily, for 5 days a week, with a total dose of 45–61.6 Gy. Subsequent high-dose-rate brachytherapy (HDR-BT) treatments were performed one week after EBRT, with a total dose range between 21 and 47 Gy at 5.6–8.6 Gy per fraction. In addition, all patients received concurrent chemotherapy with weekly cisplatin or nedaplatin at a dose of 50 mg/m^2^.

### Follow-up

Regular follow-up was conducted every third month until the 2^nd^ year after treatment, twice per year in the 3^rd^, 4^th^, and 5^th^ year, and once a year after that. Disease progression or recurrence was confirmed by gynecological examination, tumor marker measurements, and imaging modalities such as CT, MRI, and PET-CT. In addition, PFS and OS were chosen as two separate endpoints. PFS was defined as the time from the start of the treatment to disease progression, recurrence, death, or the last follow-up visit. OS was the time from the date of treatment to death by any cause or the last follow-up visit.

### Tumor image segmentation

ITK-SNAP (v.3.6.0; www.itksnap.org) was used for manual 3D segmentation of MR images. The volume of interest (VOI) of the tumor was segmented by manually drawing regions of interest (ROIs) by an experienced radiologist (X.Z. with 5 years of experience in gynecologic MR), who was blinded to the results of patients’ outcomes. The apparent diffusion coefficient (ADC) map was derived from DWI by a workstation (Advantage Workstation 4.6; GE Medical System). The ROIs were drawn along the margin of the tumor on each slice of the (i) axial oblique T2WI, (ii) sagittal arterial-phase contrast-enhanced MRI, (iii) sagittal delayed-phase contrast-enhanced MRI, and (iv) DWI images, carefully avoiding cystic, necrotic, or hemorrhagic tumor regions. The VOIs of DWI images were then finally copied into ADC maps.

To evaluate the inter-observer reproducibility of ROIs, 60 patients were randomly selected for tumor segmentation by another radiologist with 8 years of experience in gynecological imaging (Q.Z.). Subsequently, images drawn by two radiologists were analyzed for inter-group consistency.

### Radiomics feature extraction

Each MRI scan of each patient was normalized with Z-scores in order to get a standard normal distribution of image intensities, a set of 100 normalized radiomics features were extracted from each VOI segmented on each sequence by using the PyRadiomics (https://pyradiomics.readthedocs.io). These radiomics features included (i) 14 shape features, (ii) 18 first-order features, (iii) 22 gray level co-occurrence matrix (GLCM) features, (iv) 16 gray level run length matrix (GLRLM) features, (v) 16 gray level size zone matrix (GLSZM) features, and (vi) 14 gray level dependence matrix (GLDM) features. In total, 400 radiomics features were extracted from each patient. The agreement between the two radiologists was assessed by the intraclass correlation coefficient (ICC). Features with an ICC higher than 0.75 were reserved.

### Radiomics feature selection and signature construction

The predictive performance in predicting PFS and OS of each feature was evaluated by univariate Cox regression in the training group. The features with *P*-value < 0.05 were treated as significant prognostic factors and selected as candidate features. To eliminate the redundant, the correlation between the features was then calculated by Spearman or Pearson correlation analysis according to their distribution types; features with coefficient r ≥ 0.8 were removed accordingly. The least absolute shrinkage and selection operator (LASSO) Cox regression method was used to multivariate feature selection and to construct the final model. The optimal λ was selected using 5-fold cross-validation in the training group to obtain an optimal feature number and avoid over-fitting, and the feature number was therefore determined automatically by the λ. Radiomics score (Rad-score) was calculated by summing the selected features weighted by their coefficients.

### Statistical analysis

The Kolmogorov-Smirnov test examined whether the data followed a normal distribution. Levene test was used to test homogeneity of variance. The Mann-Whitney U test or independent sample t-test was used to compare the differences in the continuous variables between the training group and the testing group. Chi-squared test or Fisher’s exact test was employed for categorical variables. Univariate Cox regression analysis and LASSO Cox regression analysis were used to assess the prognostic significance of Rad-score and clinical characteristics. The predictive capability of the prediction model was evaluated using Harrell’s C-index and then presented as a nomogram. C-index of 0.50–0.70 indicates poor accuracy; 0.71–0.90 indicates moderate accuracy; > 0.90 indicates high accuracy. Cut-off values of the Rad-score and the risk score based on the combined model were obtained by Maximally Selected Test Statistics. Patients were then subdivided into low-risk and high-risk groups. Survival curves were generated using the Kaplan-Meier method, and the log-rank test was used to examine the statistical difference between curves. A likelihood ratio test was used to compare the differences of the C-index. *P*-value < 0.05 was considered statistically significant. All the statistical analyses were performed with SPSS Statistics, version 19.0, and R software, version 3.4.4.

## Results

### Patient characteristics

In the training group, the median follow-up duration was 47.7 months (range, 12.0–83.4 months). At the end of the follow-up period, tumor progression/recurrence was observed in 47 (36.7%) patients, and 29 (22.7%) patients died of persistent or recurrent disease.

In the testing group, after a median follow-up of 42.0 months (range, 6.6–80.7 months), tumor progression/recurrence was observed in 21 (36.8%) patients, and 12 (21.1%) patients died of persistent or recurrent disease. There were no differences in the clinical characteristics between the training and testing groups (*P* > 0.05, Table 1).

**Table 1:** Comparison of clinical characteristics between training and testing groups

Parameters	Training (*n* = 128)	Testing (*n* = 57)	*P*-value
Age (years, mean ± SD)	52.83 ± 8.76	52.63 ± 9.11	0.891
BMI (kg/m^2^, mean ± SD)	24.80 ± 3.44	24.63 ± 3.78	0.757
SCC-Ag (ng/mL, mean ± SD)	11.90 ± 22.20	11.02 ± 17.99	0.792
Tumor grade (%)			0.062
Low-grade (well/moderately differentiated)	81 (63.3%)	44 (77.2%)	
High-grade (poorly differentiated)	47 (36.7%)	13 (22.8%)	
T stage (%)			0.435
T2	98 (76.6%)	39 (68.4%)	
T3	25 (19.5%)	16 (28.1%)	
T4	5 (3.9%)	2 (3.5%)	
2018 FIGO stage (%)			0.928
II	64 (50.0%)	27 (47.4%)	
III	59 (46.1%)	28 (49.1%)	
IVA	5 (3.9%)	2 (3.5%)	
Tumor maximum-diameter (cm, mean ± SD)	4.49 ± 1.13	4.33 ± 1.35	0.400
LNM position (%)			0.906
Negative	74 (57.8%)	31 (54.4%)	
Pelvic LNM	40 (31.3%)	19 (33.3%)	
Para-aortic LNM	14 (10.9%)	7 (12.3%)	
LNM number (%)			0.813
0	74 (57.8%)	31 (54.4%)	
≤2	24 (18.8%)	13 (22.8%)	
>2	30 (23.4%)	13 (22.8%)	

*SD* Standard deviation, *BMI* Body mass index, *SCC-Ag* Serum levels of squamous cell carcinoma antigen, *FIGO* Federation of Gynecology and Obstetrics, *LNM* Lymph node metastasis.

### Performance of the clinical characteristics in PFS and OS prediction

Univariate Cox regression analysis for PFS and OS are shown in Table 2. The multivariate analysis showed that T stage, tumor maximum-diameter, and LNM position were independent prognostic predictors for PFS, while T stage and LNM position were independent predictors for OS (Table 2).

**Table 2:** Clinical characteristics analysis for progression-free survival and overall survival

Characteristics	Progression-free survival				Overall survival			
	Univariate analysis		Multivariate analysis		Univariate analysis		Multivariate analysis	
	HR (95% CI)	*P*-value	HR (95% CI)	*P*-value	HR(95% CI)	*P*-value	HR (95% CI)	*P*-value
Age	0.972(0.942, 1.002)	0.069			0.994(0.950, 1.041)	0.807		
BMI	0.954(0.877, 1.037)	0.265			1.003(0.895, 1.124)	0.960		
SCC	1.001(0.990, 1.012)	0.875			1.008(0.995, 1.021)	0.231		
Tumor grade	1.327(0.744, 2.366)	0.338			2.220(1.013, 4.867)	0.046		
T stage	2.934(1.952, 4410)	<0.001	1.849(1.116, 3.063)	0.017	3.332(1.897, 5.589)	<0.001	2.409(1.161, 5.002)	0.018
Tumor maximum diameter	1.508(1.178, 1.930)	0.001	1.206(0.923, 1.577)	0.170	1.610(1.171, 2.214)	0.003		
LNM position	2.476(1.666, 3.679)	<0.001	1.678(1.049, 2.684)	0.031	2.606(1.555, 4.366)	<0.001	1.521(0.758, 3.054)	0.238
LNM number	1.910(1.377, 2.650)	<0.001			2.054(1.298, 3.252)	0.002		

*BMI* Body mass index, *SCC-Ag* Serum levels of squamous cell carcinoma antigen, *LNM* Lymph node metastasis.

Clinical-predicting models were constructed based on the results. The C-index values in the training and testing groups were 0.731 and 0.725 for PFS, and 0.708 and 0.693 for OS, respectively (Table 3). In the training and testing groups, the 2018 FIGO staging system showed predictive performance with C-index values of 0.657 and 0.677 for PFS estimation (HR: 3.137; 95% CI: 1.920–5.127, *P* < 0.001), and 0.665 and 0.633 for OS estimation (HR: 3.166; 95% CI: 1.673–5.992, *P* < 0.001), respectively (Table 3). The prediction performance of the clinical-predicting model for PFS and OS estimation was significantly higher than that of the 2018 FIGO staging system (*P* < 0.05).

**Table 3:** Prognostic prediction models for the outcomes of patients with locally advanced cervical squamous cell cancer

Prediction Models	Training			Testing		
	Wald Test	*P*-value	C-index	Wald Test	*P*-value	C-index
**Progression-free survival**						
2018 FIGO staging system	20.83	<0.001	0.657	10.82	0.001	0.677
Clinical model	33.57	<0.001	0.731	15.94	<0.001	0.725
Rad-PFS	40.17	<0.001	0.764	13.62	<0.001	0.762
Combined model	50.67	<0.001	0.792	21.28	<0.001	0.809
**Overall survival**						
2018 FIGO staging system	12.54	<0.001	0.665	5.54	0.02	0.633
Clinical model	22.53	<0.001	0.708	5.96	0.01	0.693
Rad-OS	23.97	<0.001	0.793	12.72	<0.001	0.750
Combined model	31.59	<0.001	0.822	13.43	<0.001	0.785

*PFS* progression-free survival, *OS* overall survival, *FIGO* Federation of Gynecology and Obstetrics.

### Performance of the radiomic score in PFS and OS prediction

A total of twelve radiomics features were selected for Rad-score building for PFS estimation (Rad-PFS) (Table 4). Eleven radiomics features were selected for Rad-score building for OS estimation (Rad-OS) (Table 5). The features selected for the Rad-score are presented along with their calculation formulas in Supplementary Appendix E1 and E2.

**Table 4:** Radiomics features included in the construction of the Rad-PFS

	Feature name
feature 1	T2WI_original_glszm_LargerAreaLowGrayLevelEmphasis
feature 2	T2WI_original_glszm_SizeZoneNonUniformity
feature 3	ADC_original_shape_Sphericity
feature 4	ADC_original_firstorder_Kurtosis
feature 5	ADC_original_firstorder_Mean
feature 6	ADC_original_glcm_ClusterShade
feature 7	ADC_original_glcm_DifferenceVariance
feature 8	ADC_original_glcm_Imc1
feature 9	ADC_original_glcm_Idmn
feature 10	Arterial-phase_original_gldm_LowGrayLevelEmphasis
feature 11	Delayed-phase_original_firstorder_TotalEnergy
feature 12	Delayed-phase_original_glszm_ZoneEntropy

*T2WI* T2-weighted imaging, *ADC* apparent diffusion coefficient, *GLSZM* gray level size zone matrix, *GLCM* gray level co-occurrence matrix, *GLDM* gray level dependence matrix.

**Table 5:** Radiomics features included in the construction of the Rad-OS

	Feature name
feature 1	ADC_original_firstorder_Maximum
feature 2	ADC_original_glcm_ClusterProminence
feature 3	ADC_original_glcm_DifferenceVariance
feature 4	ADC_original_glcm_JointEntropy
feature 5	ADC_original_glcm_Imc1
feature 6	Arterial-phase_original_shape_Maximum2DDiameterColumn
feature 7	Arterial-phase_original_firstorder_Skewness
feature 8	Arterial-phase_original_glszm_LowGrayLevelZoneEmphasis
feature 9	Arterial-phase_original_gldm_LowGrayLevelEmphasis
feature 10	Delayed-phase_original_firstorder_TotalEnergy
feature 11	Delayed-phase_original_glrlm_RunLengthNonUniformity

*ADC* apparent diffusion coefficient, *GLCM* gray level co-occurrence matrix, *GLSZM* gray level size zone matrix, *GLDM* gray level dependence matrix, *GLRLM* gray level run length matrix.

**Fig. 2:**
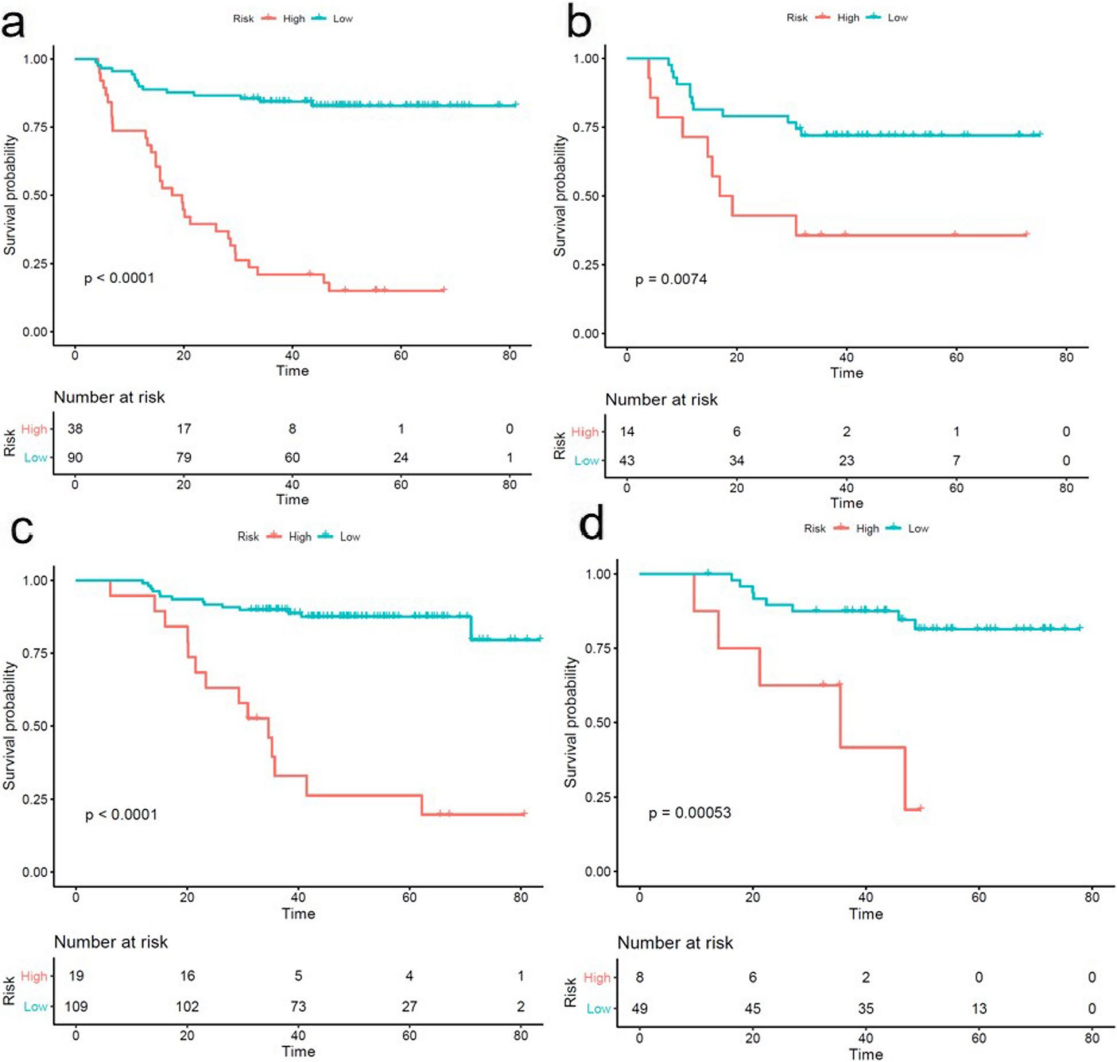
Kaplan-Meier curves of the Rad-PFS in the training group **(a)** and testing group **(b)**; Kaplan-Meier curves of the Rad-OS in the training group **(c)** and testing group **(d)**

Rad-PFS achieved significantly better predictive performance with C-index values of 0.764 and 0.762 in the training and testing groups, compared with the 2018 FIGO staging system (training and testing: *P* < 0.001) and clinical-predicting model (training: *P* = 0.037; testing: *P* < 0.001) (Table 3). Rad-OS showed predictive performance with C-index values of 0.793 and 0.750 in the training and testing groups, which were significantly higher than those of the 2018 FIGO staging system (training and testing: *P* < 0.001) and clinical-predicting model (training: *P* = 0.002; testing: *P* < 0.001) (Table 3). Patients were then further divided into high-risk and low-risk groups according to the cut-off values of Rad-PFS (0.56) and Rad-OS (1.12). The Kaplan-Meier curves for PFS and OS are shown in Fig. 2**.** Patients with the lower value of Rad-PFS or Rad-OS had significantly longer PFS and OS in the training and testing groups (*P* < 0.05).

### Performance of the combined models in PFS and OS prediction

For PFS estimation, the further multivariable Cox regression analyses incorporating the Rad-score and clinical characteristics identified Rad-PFS (HR: 2.201; 95% CI: 1.570–3.084, *P* < 0.001), T stage (HR: 1.390; 95% CI: 0.806–2.298, *P* = 0.237), and LNM position (HR: 1.435; 95% CI: 0.906–2.273, *P* = 0.124) as independent predictors. The combined model achieved the best predictive performance with C-index values of 0.792 and 0.809 in the training and testing groups, respectively, which were significantly higher than those of the single Rad-PFS (training: *P* = 0.002; testing: *P* < 0.001), clinical-predicting model (training and testing: *P* < 0.001), and 2018 FIGO staging system (training and testing: *P* < 0.001) (Table 3).

For OS estimation, the further multivariable Cox regression identified Rad-OS (HR: 2.297; 95% CI: 1.428–3.696, *P* < 0.001), T stage (HR: 1.564; 95% CI: 0.715–3.424, *P* = 0.263), and LNM position (HR: 1.321; 95% CI: 0.631–2.765, *P* = 0.461) as independent predictors. The combined model also achieved the best discrimination performance with C-index values of 0.822 and 0.785 in the training and testing groups, respectively, which were significantly higher than those of the single Rad-OS (training: *P* = 0.041; testing: *P* < 0.001), clinical-predicting model (training and testing: *P* < 0.001), and 2018 FIGO staging system (training and testing: *P* < 0.001) (Table 3).

**Fig. 3:**
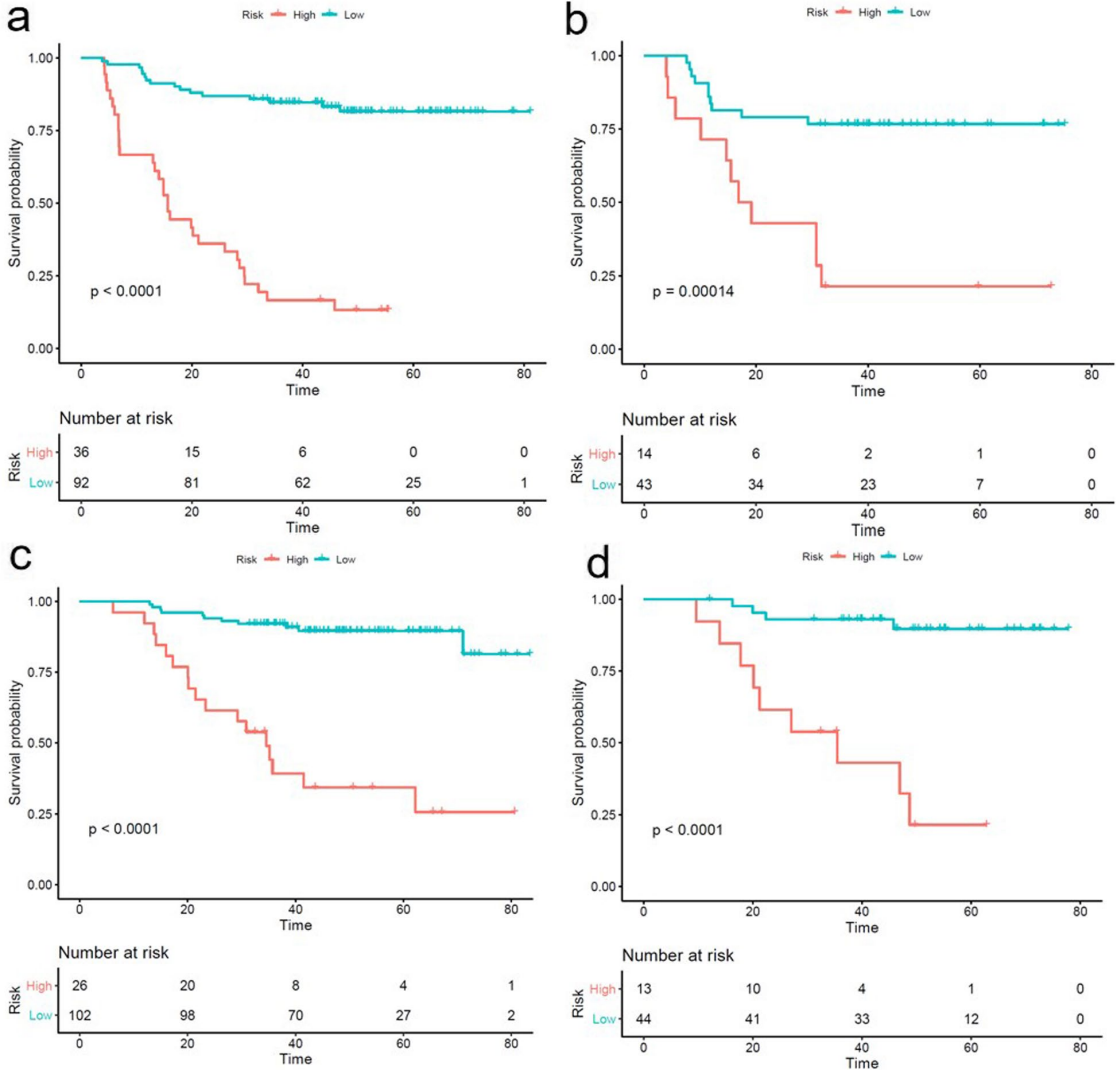
Kaplan-Meier curves of the combined model for PFS in the training group **(a)** and testing group **(b)**; Kaplan-Meier curves of the combined model for OS in the training group **(c)** and testing group **(d)**

**Fig. 4:**
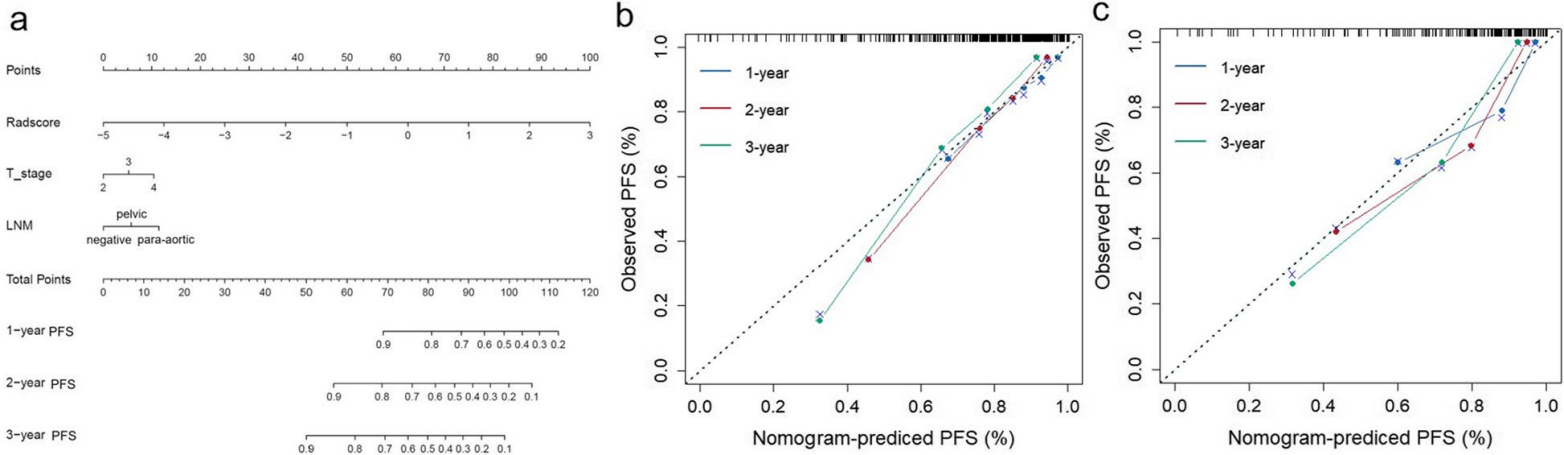
The nomogram **(a)** and calibration curves of training group **(b)** and testing group **(c)** for the combined PFS prediction model. The diagonal dashed line represents a perfect prediction by an ideal model. The blue, red, and green line represents the performance of the nomogram, of which a closer fit to the diagonal line represents a better prediction

**Fig. 5:**
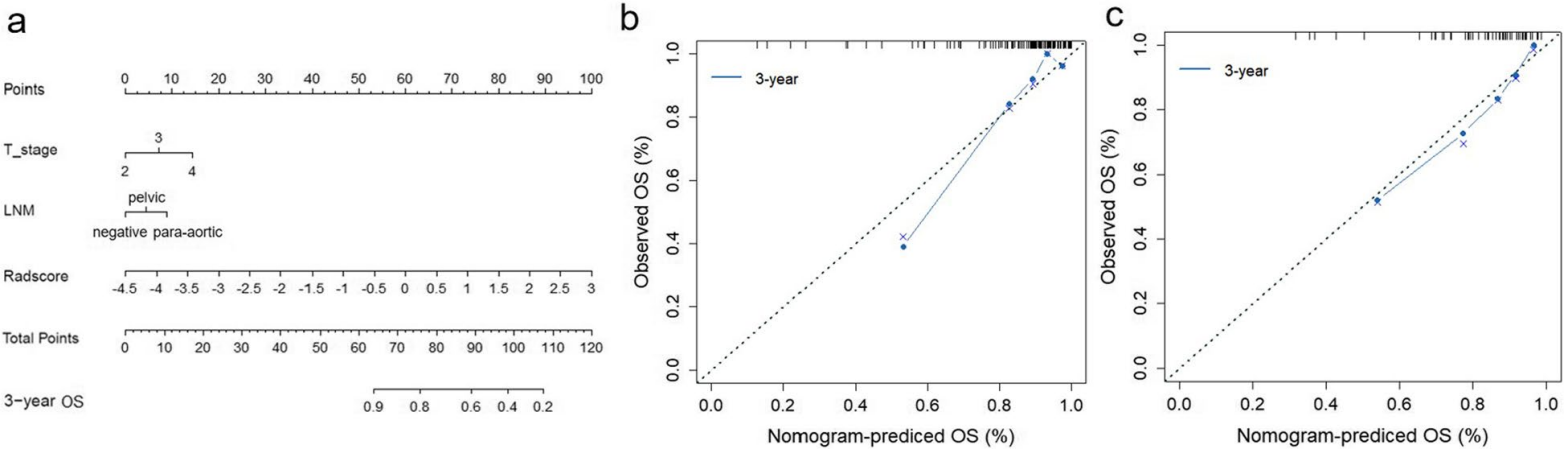
The nomogram **(a)** and calibration curves of training group **(b)** and testing group **(c)** for the combined OS prediction model. The diagonal dashed line represents a perfect prediction by an ideal model. The blue line represents the performance of the nomogram, of which a closer fit to the diagonal line represents a better prediction

The calculation formulas of the risk scores were shown in Supplementary Appendix E3 and E4. Patients were then subdivided into low-risk and high-risk groups according to the cut-off values of the risk scores of the combined models for PFS (1.44) and OS (1.95); the Kaplan-Meier curves for PFS and OS of the combined model are shown in Fig. 3. Patients with the lower risk score value had significantly longer PFS and OS in the training and testing groups (*P* < 0.05). The nomograms and calibration curves of the combined models for PFS and OS are shown in Fig. 4 and Fig. 5, respectively.

## Discussion

This study developed and validated Rad-score from MRI for prognosis estimation in patients with LACSC treated with chemoradiotherapy. The MRI-based Rad-score showed moderate accuracy in PFS and OS prediction, which was more accurate than the 2018 FIGO staging system and the clinical model. The combined model, including Rad-score and clinical characteristics, showed the best prediction performance in PFS and OS prediction. Moreover, patients were subdivided into low-risk and high-risk groups according to the risk score of the combined model, which can further facilitate individualized PFS and OS estimation prior to initiation of chemoradiotherapy.

Until 2018, cervical cancer was the only gynecologic malignancy staged primarily based on clinical findings. Importantly, LNM is not part of the previous FIGO system, despite LNM is one of the most important prognostic indicators for recurrence and death in patients with cervical cancer [21]. In 2018, the FIGO classification was revised, further incorporating imaging and pathologic findings, and allowing the addition of LNM. The new 2018 FIGO staging system has a better stage differentiation and predictive accuracy for disease-free survival (DFS) compared to the 2014 FIGO staging system [22]. However, the modified 2018 FIGO staging system is still under evaluation and needs to be further improved. This study pointed out that the discrimination of the 2018 FIGO staging system for PFS and OS is still not visually ideal. Our results showed that the LNM was the independent prognostic factor for PFS and OS; the patients with LNM had worse PFS and OS, especially those with para-aortic LNM. We also found that a higher T stage is correlated with worse PFS and OS, which was consistent with previous studies [23–25], indicating that local tumor factors remain salient prognostic factors in cervical cancer. Moreover, Mu et al [26] found that the clinical-predicting model including T stage and LNM for PFS and OS had a significantly higher C-index than the 2018 FIGO staging system in the patients with LACC, which was consistent with our study. However, the clinical-predicting model lacks features reflecting tissue microstructure. Currently, the intratumor heterogeneity has been reported to have pronounced effects on the prognosis of tumor [27]. Further study about it may be helpful in the more precise prediction of PFS and OS in patients with LACSC treated with CCRT.

The radiomics approach could noninvasively extract useful imaging features from medical images, which may help reflect the intratumor heterogeneity and provide underlying diagnostic, therapeutic, and prognostic information [28]. Preliminary reports suggested a potential use of radiomics analysis in cervical cancer imaging. The value of MRI radiomics model has been proven in predicting the LNM and lymph-vascular space invasion status in patients with cervical cancer preoperatively [29, 30]. Becker et al [31] reported that the textural parameter of the ADC map correlates with the differentiation of cervical cancer. Giving this background, radiomics features based on MRI were used to predict the survival of cervical cancer patients in recent years. Wormald et al [32] found that radiomics features from ADC maps and T2WI could potentially predict recurrence in patients with stage I-II low-volume cervical cancer. Moreover, Laliscia et al [19] found that the radiomics features from T2WI are useful for predicting the prognosis of LACC. In addition, a retrospective study of 248 stage IB–IIA cervical cancer patients investigated the prognostic value of the pretreatment MRI (T2WI and contrast-enhanced T1WI) based Rad-score for DFS estimation [33], and 18 radiomics features were identified to be predictive for DFS, including 10 features derived from contrast-enhanced T1WI and 8 features extracted from T2WI; the Rad-score yielded a C-index of 0.753 on DFS prediction. This may suggest that contrast-enhanced T1WI probably contains more prognostic information than T2WI and that MRI-derived Rad-score can be used as a prognostic biomarker for patients with early-stage cervical cancer.

This study used radiomics features from multi-parametric MRI to establish the Rad-score. The multi-parametric MRI could reduce the risk of bias of the radiomics features obtained from a single sequence. We included the multi-phase contrast enhanced MRI (arterial-phase and delayed-phase) in addition to T2WI and ADC, different sequences could reflect different characteristics of tumors, including tumour intensity, cellularity and vascularisation [34]. ADC map with a reflection of the biological heterogeneity of tumors can provide better characterization of tissue and their pathological processes at the microscopic level [35]. The multi-phase contrast enhanced MRI may reflect intratumoral heterogeneity and architecture (e.g., tumor angiogenesis) and the changes in the tumor’s blood supply, while T2WI can detect tumor density [36]. Most locally advanced cervical tumors present with high intratumoral heterogeneity in virtually all distinguishable phenotypes, such as proliferation, vascularity, metabolism, oxygenation, etc., which directly suggests tumor resistance to therapy and poor prognosis [37, 38]. Our study revealed that the prediction performance of the Rad-PFS and Rad-OS was better than that of the clinical-predicting models, and the combination of Rad-score and clinical features can achieve the best prediction performance, indicating that radiomics can mine more prognostic information than clinical factors by observing the whole tumor scope and extracting high-dimensional features. Thus, it could be used as a surrogate biomarker to improve the prognostic ability pretreatment.

Despite the favorable results of MRI-based radiomics, this study has several limitations. First, this study was a single-center and retrospective study. Thus, further validation of the prediction model based on external centers and large-scale cohorts is required. Second, the follow-up was relatively short; a longitudinal study is needed to further evaluate the long-term prognostic value of MRI-based radiomics analysis in LACSC. Third, the selection criteria of LNM might lead to the exclusion of patients with smaller LNM or a false positive of the included LNM, it is hoped to plan a prospective study in patients with a pathological assessment of LNM in the future.

## Conclusions

In conclusion, this study suggests that MRI-based Rad-score could provide effective information for predicting the outcomes of LACSC treated with CCRT. Compared with the commonly used 2018 FIGO staging system and clinical characteristics, the Rad-score could significantly improve the prediction performance. The combined model (including Rad-score and clinical characteristics) showed the best prediction performance. The patients stratified by the combined model can be classified into low-risk and high-risk groups for PFS and OS, which might provide clinicians with new insights into individualized follow-up and guiding therapeutic strategies.

## Supplementary Information

**Additional file 1.**

## Data Availability

The datasets used and/or analysed during the current study are available from the corresponding author on reasonable request.

## Abbreviations

MRI: Magnetic resonance imaging
ADC: Apparent diffusion coefficient
DWI: Diffusion-weighted imaging
LACC: Locally advanced cervical carcinoma
LACSC: Locally advanced cervical squamous cell cancer
CCRT: Concurrent chemoradiotherapy
FIGO: Federation of Gynecology and Obstetrics
LNM: Lymph node metastasis
BMI: Body mass index
SCC-Ag: Squamous cell carcinoma antigen
OS: Overall survival
PFS: Progression-free survival
DFS: Disease-free survival
ROI: Region of interest
VOI: Volume of interest
EBRT: External pelvic beam radiation therapy
HDR-BT: High-dose-rate brachytherapy
ICC: Interclass correlation coefficient
LASSO: Least absolute shrinkage and selection operator
