# Supplementary material for: MRI-based radiomics value for predicting the survival of patients with locally advanced cervical squamous cell cancer treated with concurrent chemoradiotherapy
Source: Cancer Imaging. 2022 Jul 16;22:35. doi: 10.1186/s40644-022-00474-2 (PMC9287951; doi:10.1186/s40644-022-00474-2)
Supplement: Supplementary file 1 — Additional file 1. [file 40644_2022_474_MOESM1_ESM.docx]

**Table S1** MR imaging parameters

| Sequence | Imaging Plane | TR(ms)/TE(ms) | Slice  Thickness(mm) | Gap  (mm) | Field of View(mm) | Acquisition matrix  (phase × frequency) | Number of Excites | b-values  (sec/mm^2^) |
| --- | --- | --- | --- | --- | --- | --- | --- | --- |
| **GE signa excite HD 3.0T** |  |  |  |  |  |  |  |  |
| FS FSE pelvic T2WI | Axial | 5400/106.5 | 5 | 1 | 400 | 320*256 | 2 | - |
| FS FSE retroperitoneal T2WI^a^ | Axial | 4900/100.2 | 5 | 1 | 400 | 320*256 | 2 | - |
| FSE T2WI | Axial oblique | 5200/134.6 | 4 | 1 | 220 | 320*256 | 1 | - |
| DWI | Axial | 4700/64.4 | 5 | 1 | 400 | 128*128 | 2 | 0, 800 |
| Multi-phase contrast enhanced MRI | Sagittal | 3.8/1.8 | 3 | 0 | 300 | 320*192 | 0.7 | - |
| **GE Discovery HD750 3.0T** |  |  |  |  |  |  |  |  |
| FS FSE pelvic T2WI | Axial | 4734/90.3 | 5 | 1 | 380 | 320*256 | 2 | - |
| FS FSE retroperitoneal T2WI^a^ | Axial | 4734/87.9 | 5 | 1 | 380 | 320*256 | 2 | - |
| FSE T2WI | Axial oblique | 6241/121.6 | 4 | 0.4 | 200 | 320*256 | 1 | - |
| DWI | Axial | 4000/56 | 5 | 1 | 380 | 128*128 | 6 | 0, 800 |
| Multi-phase contrast enhanced MRI | Sagittal | 3.8/1.8 | 3 | 0 | 340 | 320*192 | 0.7 | - |

^a^Retroperitoneal T2WI sequence was performed from the renal hilum level to the first slice of the pelvic axial T2WI sequence. Axial oblique T2WI, multi-phase contrast enhanced MRI, and DWI were used to the extraction of radiomics features. Retroperitoneal T2WI and pelvic axial T2WI were used to evaluate the retroperitoneal and pelvic lymph node status.

*FSE* fast spin echo, *FS* fat suppression, *TR* repetition time, *TE* echo time, *T2WI* T2-weighted imaging, *DWI* diffusion-weighted imaging, *MRI* magnetic resonance imaging

**Appendix E1**. Radiomics score (Rad-score) calculation formula for PFS estimation

Rad-PFS = 0.005 * T2WI_original_glszm_LargerAreaLowGrayLevelEmphasis + 0.059 * T2WI_original_glszm_SizeZoneNonUniformity - 0.283 * ADC_original_shape_Sphericity + 0.133 * ADC_original_firstorder_Kurtosis - 0.105 * ADC_original_firstorder_Mean - 0.590 * ADC_original_glcm_ClusterShade - 0.342 * ADC_original_glcm_DifferenceVariance + 0.134 * ADC_original_glcm_Imc1 + 0.243 * ADC_original_glcm_Idmn + 0.314 * Arterial-phase_original_gldm_LowGrayLevelEmphasis + 0.055 * Delayed-phase_original_firstorder_TotalEnergy + 0.117 * Delayed-phase_original_glszm_ZoneEntropy

**Appendix E2**. Radiomics score (Rad-score) calculation formula for OS estimation

Rad-OS = -0.114 * ADC_original_firstorder_Maximum - 0.502 * ADC_original_glcm_ClusterProminence - 0.426 * ADC_original_glcm_DifferenceVariance - 0.127 * ADC_original_glcm_JointEntropy + 0.211 * ADC_original_glcm_Imc1+0.211 * Arterial-phase_original_shape_Maximum2DDiameterColumn + 0.094 * Arterial-phase_original_firstorder_Skewness + 0.456 * Arterial-phase_original_glszm_LowGrayLevelZoneEmphasi + 0.093 * Arterial-phase_original_gldm_LowGrayLevelEmphasis + 0.354 * Delayed-phase_original_firstorder_TotalEnergy + 0.008 * Delayed-phase_original_glrlm_RunLengthNonUniformity

**Appendix E3**. Risk score calculation formula for PFS estimation

Risk score(PFS) = 0.329 * T stage + 0.361 * LNM position + 0.789 * Rad-PFS

**Appendix E4**. Risk score calculation formula for OS estimation

Risk score(OS) = 0.447 * T stage + 0.278 * LNM position + 0.832 * Rad-OS
